# Carrying Position Independent User Heading Estimation for Indoor Pedestrian Navigation with Smartphones

**DOI:** 10.3390/s16050677

**Published:** 2016-05-11

**Authors:** Zhi-An Deng, Guofeng Wang, Ying Hu, Yang Cui

**Affiliations:** 1 School of Information Science and Technology, Dalian Maritime University, Dalian 116026, China; gfwangsh@163.com (G.W.); huying@dlmu.edu.cn (Y.H.); 2 School of computer science and technology, Harbin Institute of Technology, Harbin 150001, China; ycui@hit.edu.cn

**Keywords:** heading estimation, carrying position, inertial sensors, principal component analysis

## Abstract

This paper proposes a novel heading estimation approach for indoor pedestrian navigation using the built-in inertial sensors on a smartphone. Unlike previous approaches constraining the carrying position of a smartphone on the user’s body, our approach gives the user a larger freedom by implementing automatic recognition of the device carrying position and subsequent selection of an optimal strategy for heading estimation. We firstly predetermine the motion state by a decision tree using an accelerometer and a barometer. Then, to enable accurate and computational lightweight carrying position recognition, we combine a position classifier with a novel position transition detection algorithm, which may also be used to avoid the confusion between position transition and user turn during pedestrian walking. For a device placed in the trouser pockets or held in a swinging hand, the heading estimation is achieved by deploying a principal component analysis (PCA)-based approach. For a device held in the hand or against the ear during a phone call, user heading is directly estimated by adding the yaw angle of the device to the related heading offset. Experimental results show that our approach can automatically detect carrying positions with high accuracy, and outperforms previous heading estimation approaches in terms of accuracy and applicability.

## 1. Introduction

Though global navigation satellite systems (GNSS) may provide accurate localization outdoors, there is still no equivalent dominant indoor positioning technique. Most existing technologies, such as ultra-wideband [1], and radiofrequency identification (RFID) [2], require dedicated hardware, which is expensive for large scale deployment and thus always not seamlessly available during pedestrian walking. WiFi localization [3] can exploit the existing wireless infrastructures and ubiquitously available network cards on a smartphone. However, tedious data collection effort [4] is required to map a complex indoor wireless signal propagation environment. Recently, pedestrian dead reckoning (PDR) [5] using the inertial sensors built into a smartphone has been considered as a promising candidate for seamless indoor navigation. The widescale use of smartphones in our daily lives and their self-contained inertial sensors make smartphones ideal devices for low-cost and continuous indoor navigation.

The key challenge for PDR using a smartphone [6] is the user heading estimation. While the user heading, step counting and step length are estimated, the user’s current location can be determined by adding the relative displacement to the previous location. Assuming that the initial user location is known, if the heading estimation problem is well solved, a continuous indoor pedestrian tracking may be achieved by PDR. In fact, since the user heading problem can be transformed into a user facing direction problem, it has also attracted extensive interest from many other application areas, such as behavioral analysis [7], and human computer interactions [8]. Compared with heading estimation depending on dedicated ambient sensors [9] or intrusive wearable sensors [10], smartphones using inertial sensors provide a more natural and less intrusive heading estimation solution.

Many heading estimation approaches using a smartphone only consider a simplified situation, where both the carrying position and device attitude are specified and fixed [11,12]. This situation is true when a smartphone is held in hand. Under this situation, once the heading offset between the device forward direction and the user heading is known, the user heading can be directly determined by a device attitude estimation approach [13,14]. However, this approach is inapplicable when the smartphone is put in a trouser pocket, a more likely carrying position, especially for young males [15]. Under this highly dynamic situation, the heading offset is varying all the time with the change of device attitude relative to the user’s body. Recently, to address the heading estimation problem for the trouser pocket situation, the conventional PCA approach [16], our previous proposed RMPCA approach [17] combining rotation matrix (RM) and PCA, and the uDirect approach [18] have been proposed.

For the RMPCA approach, PCA is firstly utilized to extract the local walking direction in the device coordinate system; then the global walking direction is obtained by mapping the local walking direction into the global coordinate system using a rotation matrix between the two coordinate systems. RMPCA approach is independent of device attitude, since the changing device coordinate system caused by leg locomotion is accurately tracked and computed by the rotation matrix.

Despite the potential of smartphone-based heading estimation solutions, several critical problems still need to be addressed to enhance the accuracy and applicability of heading estimation to daily use situations. Firstly, the current heading estimation solutions are constrained to a particular carrying position. There is no selection scheme for optimal heading estimation approach of different carrying positions. For PCA-based approaches, for example, whether they are applicable to other carrying positions has not been clearly analyzed. Different carrying positions during pedestrian walking may render different performances of PCA-based approaches.

Secondly, in order to achieve selection of an optimal heading estimation approach, an automatic recognition of carrying position is required. Requiring a user to manually provide information about the carrying position makes the system more intrusive, especially when the carrying positions change frequently. Furthermore, the position recognition technique should capture carrying position transitions as fast as possible while adding little computation cost on resource limited smartphones.

Thirdly, the motion state should be predetermined before carrying position recognition and heading estimation. A carrying position classifier designed for a specific motion state renders a more compact input feature space, which is beneficial for improving classification accuracy. Moreover, motion states may directly impact the user heading estimation. For example, the user heading when using an escalator is always limited to a small specific region, which can be used to recalibrate the user heading.

In this work, we present a novel heading estimation system independent of carrying position. The proposed system includes three major phases. The first phase uses accelerometers and barometers to predetermine the motion state of a pedestrian, including standing, walking, walking upstairs and downstairs, taking elevators and escalators. The second phase recognizes the carrying position of a smartphone automatically. We develop a position recognition classifier using a feature set generated by acceleration signals. Moreover, little attention has been paid to the confusion caused by user turns and carrying position transitions, which may render a large heading estimation error. We develop a position transition detection algorithm to address this problem, and combine it with a position classifier to reduce the computational cost of position recognition.

With the recognized carrying position, the third phase selects the most appropriate heading estimation strategy. It allows a more accurate heading estimation than any generic approach adopted. For positions in the trouser pocket (in-pocket) or swinging in hand (swing-hand), we deploy a RMPCA-based approach. For positions held in hand (hand-held) or against ear during phone calls (phone-call), we achieve heading estimation by adding the yaw angle of the device and the related heading offset, which may be reset immediately after a carrying position transition occurs.

Experiments are carried out by four participants on a smartphone to verify the performance and show that our heading estimation system performs more accurately and reliably than existing individual approaches. In summary, our work makes the following contributions:

- We propose a novel system for user heading estimation independent of carrying position.
- We develop a computationally lightweight carrying position recognition technique that performs accurately using acceleration signals.
- We propose a novel carrying position transition detection algorithm, which may be used to distinguish between user turns and position transitions, and reduces the computational cost of position recognition.
- We further examine the application of PCA-based approaches for the other carrying positions and develop an extended Kalman filter-based attitude estimation model for heading estimation.
- We report the evaluation of the proposed position recognition technique based on extensive samples collected from four participants and compare the performance of our heading estimation approach to existing approaches.

In the rest of this paper, we will firstly discuss the related works in Section 2. Section 3 introduces an overview of the proposed heading estimation system. Section 4 and Section 5 describe the motion state and position recognition techniques in detail, respectively. Section 6 describes the heading estimation approaches optimized for different carrying positions. The experimental results are reported and discussed in Section 7. Conclusions and future works are presented in the last section.

## 2. Related Works

Compared with many dedicated wearable sensors, including infrared ray transceivers [9], stereo cameras [10], and NavShoe [19] that are mounted on fixed positions with fixed orientations, smartphone inertial sensors for heading estimation [20] are more practical and less intrusive for daily life use. No additional hardware cost is required and their carrying positions and orientations may be selected flexibly. Most existing smartphone-based user heading estimation [21] approaches assume that the carrying position and orientation are fixed throughout their operation. For example, the smartphone is always assumed to be held in hand and so that traditional attitude estimation-based approaches may be applicable for heading estimation. However, for a smartphone freely placed in other positions during pedestrian walking, such as in a trouser pocket, the assumption is seriously corrupted by the changing device attitude due to the highly dynamic body locomotion. As a result, the smartphone attitude estimation-based heading estimation approach is inapplicable for in-pocket carrying positions, since the heading offset between the yaw angle of the device and the user heading may vary with body locomotion.

Hoseinitabatabaei *et al.* [18] have proposed an uDirect approach for heading estimation using a smartphone placed in the user’s trouser pocket. They extract the user’s walking direction directly within a specific region, where the horizontal acceleration plane is dominated by forward acceleration. Unfortunately, such a region is always corrupted by sideway acceleration components due to the body locomotion dynamics.

Kunze *et al.* [16] have deployed PCA for heading estimation using a smartphone put in the user’s trouser pocket. The first principal component of PCA over the horizontal plane of acceleration signals is assumed to be parallel to the user’s walking direction. Steinhoff *et al.* [22] have performed an experimental study of such PCA-based techniques, and further introduce proper filtering of acceleration signals to improve the heading estimation accuracy. In order to avoid the inaccurate estimation of the horizontal acceleration plane caused by changing device coordinates, our previous work has proposed the RMPCA approach, which combines PCA with a rotation matrix that may track the device coordinates accurately and continuously. However, all the PCA-based approaches are specifically designed for the trouser pocket position, whether they are suitable for other positions is unclear and needs further analysis. Even though the PCA-based approach is optimal for some other positions, an automatic position recognition technique is required, since requiring the user to manually set the carrying position is impractical.

Recently, Shoaib *et al.* [23] have studied the performance of various motion sensors or their combinations in recognizing different physical activities with various carrying positions. However, the classifier is not designed for position recognition during pedestrian walking, and some typical carrying positions for pedestrian walking, such as swinging in the hand and against the ear during phone calls, are still not considered. More importantly, it is necessary to distinguish between position transitions and user turns during pedestrian walking, since confusing the two motions will render a large heading estimation error.

In order to overcome the aforementioned shortcomings of previous works, this work proposes a novel heading estimation system independent of carrying position. We firstly automatically recognize the carrying positions by combining a position classifier with a position transition detection algorithm, and then select the most suitable heading estimation strategy for each carrying position. Experimental results show that the proposed heading estimation approach achieves significant accuracy improvement over existing individual approaches.

## 3. System Overview

**Figure 1:**
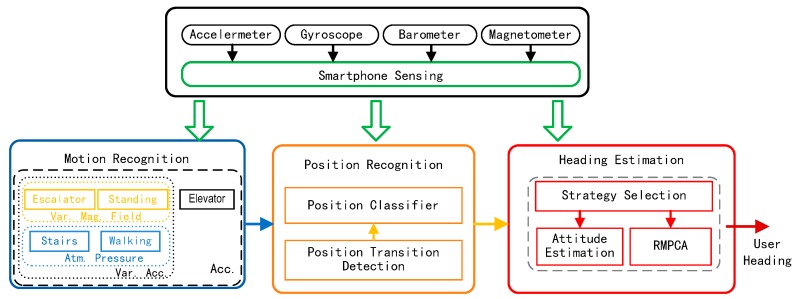
Overview of the proposed user heading estimation system.

Figure 1 shows an overview of the proposed user heading estimation system, which includes four main stages: sensing, motion recognition, position recognition and heading estimation stages. The sensing stage continuously collects samples from the accelerometer, gyroscope, and barometer sensors built into a smartphone. Barometer signals are only employed by the motion recognition stage to identify the user motion state involving vertical displacements.

The motion recognition stage deploys a decision tree-based approach to identify the motion state of a user. A predetermined motion state may directly impact the heading estimation. When using escalators or walking out of or entering elevators when taking elevators, the user heading is always limited into a small specific region, which can be used to recalibrate the user heading. For standing state, the user heading remains unchanged. For walking, walking upstairs/downstairs states, the most appropriate heading estimation strategy is selected according to the proposed selection algorithm in the third stage. For simplicity, the details of the process for exploring particular motion states to recalibrate the heading estimation and related experiments will be given in our future work.

Based on the determined motion state, the position recognition stage deploys a carrying position classifier to map the measured acceleration data to a set of predefined positions. Unlike previous position recognition techniques requiring a continuously working position classifier, we only require it to work at the start of the application and each time a carrying position transition occurs. To detect the position transition, we develop a novel computationally lightweight detection algorithm.

The recognized positions are then updated and fed into the heading estimation stage for selecting the most suitable heading estimation strategy: an attitude estimation-based approach or the RMPCA approach. Since both approaches require the device attitude information, we develop an extended Kalman filter (EKF)-based device attitude model by deploying a rotation quaternion.

## 4. Motion State Recognition

**Figure 2:**
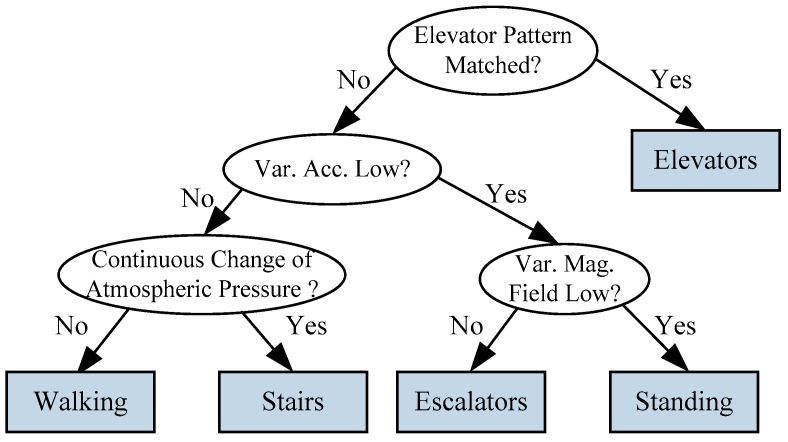
Motion state recognition using a decision tree.

We firstly predetermine the user motion state through a decision tree, as seen in Figure 2. In this work, we investigate different motion states including standing, walking, taking elevators and escalators, and walking upstairs/downstairs. The sensors deployed in identification of these motion states are accelerometers, barometers and magnetometers.

As shown in Figure 2, in the first level of decision tree, we distinguish the motion state of taking elevators from the other states based on its unique acceleration pattern [24]. A typical elevator usage trace includes normal walking state, followed by a standing state for some time to wait for the elevator, walking into the elevator, standing inside for a short time, a hyper-/hypo-gravity process (depending on the direction of the elevator), then a stationary period which depends on how many floors the elevator moved, another hypo-/hyper-gravity process, and finally a walk-out.

In the second level of the decision tree, variance of acceleration signals is explored to distinguish between walking/walking on stairs and taking escalators/standing, since the former pair of motion states involves much higher intensity of body locomotion than the latter one. In the third level of the decision tree, variance of the magnetic field can be a reliable feature to distinguish between taking escalators and standing. This is because the variance of magnetic field of taking escalators is significantly larger than that of standing due to the motor of escalators.

To further separate normal walking from walking upstairs/downstairs, atmospheric pressure from a barometer is explored. It is known to all that the higher the elevation, the lower the atmospheric pressure [25]. Therefore, a sum of absolute differences between adjacent atmospheric pressure samples within a sliding window is deployed as a flag. If the flag exceeds a threshold, walking upstairs or downstairs are identified. To smooth instantaneous measurement noise, one atmospheric pressure sample is generated by averaging raw measured samples over one second with a sampling frequency of 20 Hz.

It should be noted that the indoor atmospheric pressure on the same floor may vary due to various factors such as opening/closing of windows or the operation of air conditioning systems. Fortunately, except for a sudden weather change, the changes of atmospheric pressure caused by most of these factors are always significantly smaller than those caused by walking upstairs/downstairs during a short period. For example, according to our experiments, the walking upstairs/downstairs flag may reach more than 30 Pa within a sliding window of eight seconds with vertical displacements of about three meters, while that of standing two meters away from a window transiting from closed to open state is only about 10 Pa during the same time. Therefore, an appropriate choice of threshold will avoid false detection of walking upstairs/downstairs caused by most atmospheric pressure perturbations.

Through the decision tree developed for motion recognition, the average classification accuracy can reach about 99%. Therefore, we assume that the motion sate may be correctly predetermined in the subsequent carrying position recognition stage of the heading estimation system.

## 5. Position Recognition

In order to select an optimal heading estimation strategy, it is crucial to recognize the device position on a user’s body in an accurate and timely way. In this work, we investigate the most four common carrying positions during pedestrian walking [26,27]: hand-held, phone-call, in-pocket and swinging-hand. In Section 5.1, we firstly present a carrying position classifier designed for a specific motion state. In Section 5.2, to reduce the computational cost, we develop a novel computationally lightweight position transition detection algorithm to continuously monitor position transitions. In Section 5.3, the position transition detection is also explored to discriminate between user turns and position transitions.

### 5.1. Carrying Position Classifier

For a multi-class carrying position classifier, it is beneficial for enhancing the classification accuracy to design the classifier based upon a specific motion state. This is because different motion states show different acceleration signal patterns [27], and the classifier designed based upon a specific motion state may render a more compact input feature space, which is useful to avoid overfitting problems during carrying position classification. Under a specific user motion, such as walking on level ground, we collect acceleration signals with different carrying positions. Then, the collected data are divided into small segments for feature extraction by a sliding window. As previous studies suggested [23], we select a window size of two seconds and with 50% overlap between consecutive windows, which have been shown to produce a reasonable position recognition performance.

Once the data pre-processing is completed, features are extracted from acceleration signals within the sliding window. If the classifiers are trained only for a specific orientation, the recognition performance may degrade due to the orientation changes caused by body locomotion, especially when a smartphone is placed in a trouser pocket. In order to mitigate the negative effect caused by orientation changes, except for training the classifier upon the same carrying position with different device orientations, we add the magnitude of acceleration signal as a fourth dimension of existing three dimensional acceleration signals. The magnitude of acceleration signals is less sensitive to orientation changes, which may enhance the generalization ability of the position classifier. All acceleration signals are measured at device coordinate system (DCS), which is defined by axes *X_D_*, *Y_D_*, and *Z_D_*. *X_D_* and *Y_D_* are both parallel to the smartphone screen, and point rightward and forward, respectively. *Z_D_* is the cross product of *X_D_* and *Y_D_*.

**Figure 3:**
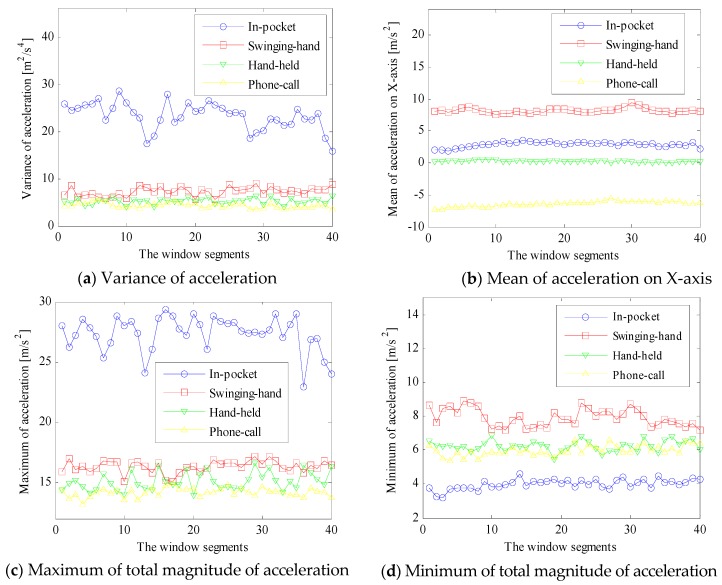
Feature comparisons with different carrying positions.

Based on the four dimensional signal, we select mean, variance, maximum and minimum as features which are computationally cheap and effective to distinguish different carrying positions. The basic principle for exploring the four features to distinguish different carrying positions is that, the magnitude and variance of accelerations are significantly larger for in-pocket and swinging-hand positions than those of hand-held and phone-call positions, due to the higher intensity of body locomotion. This principle is true, regardless of device orientation changes. Mean is defined as the average value of all samples in the window segment, and variance is defined as the average squared deviation of all sample values from its mean in the window segment. Maximum and minimum are the maximal and minimal acceleration signals of each dimension in the window segment, respectively. Figure 3a shows an example of the variances of the magnitude of accelerations with different carrying positions during pedestrian walking. The in-pocket acceleration variance is the largest and much larger than those of other carrying positions, due to the highly dynamic leg locomotion. The swinging-hand acceleration variance is the second largest, while those of hand-held and phone-call positions are confused and cannot be distinguished from each other clearly.

Figure 3b shows an example of mean values of acceleration on the *X*-axis of a smartphone (*X_D_*) with different carrying positions. Mean values of the measured accelerations on the *X*-axis include two parts: body accelerations on the *X*-axis and a sub-component of the gravity vector on the *X*-axis. The first part may be averaged and reduced to a small value due to the periodic acceleration and deceleration of body locomotion. Therefore, mean values are mainly determined by the sub-component of the gravity vector on the *X*-axis. For the hand-held position, the direction of the *X*-axis is usually aligned with the right side of the user’s body, which is almost orthogonal to the gravity vector and walking direction. Therefore, the related mean acceleration on the *X*-axis is near zero when a smartphone is held in the hand during normal walking. For a swinging-hand position, the mean acceleration on the *X*-axis is near to the gravity value, since the direction of the *X*-axis is almost aligned with the opposite direction of the gravity vector. For in-pocket and phone-call positions, the related mean values of the acceleration on the *X*-axis are positive and negative, respectively, with a smaller magnitude than that of gravity vector. This is because a negative and positive sub-component of the gravity vector is added on the *X*-axis, respectively. In fact, not only the mean values of acceleration on *X*-axis, but also on the other two axes may distinguish between different carrying positions to some extent. This phenomenon also motivated us to deploy the change of acceleration signals over three dimensions to detect the position transitions, as shown in the next subsection.

Figure 3c,d show an example of the maximum and minimum of the total magnitude of accelerations with different carrying positions, respectively. For different carrying positions, the maximum features increase with the intensity of physical movement. Maximum features of an in-pocket position are the largest, and those of swinging-hand are significantly larger than those of hand-held and phone-call positions. In contrast, the minimum features of in-pocket are the smallest, while those of swinging-hand are the largest, due to the different cancelling effects of the gravity vector and body acceleration.

After feature extraction, we select three classical classifiers to test the position classification accuracy: Support Vector Machine (SVM) [28], Bayesian Network [29] and Random Forest [30]. Extensive samples from four participants with different carrying positions are collected and used to train the classifiers. Experimental results show that different carrying positions can be classified with high accuracy. The corresponding results and detailed analysis are provided in Section 7.

### 5.2. Carrying Position Transition Detection Algorithm

Unlike previous position recognition techniques [23] continuously performing a position classifier to recognize and update the device carrying position, we monitor the carrying position by a novel position transition detection algorithm. The basic idea to perform a position transition detection process is that the probability of a position transition for pedestrian smartphone daily use is always small, and thus a computationally cheap and effective position transition detection algorithm is sufficient for almost all cases. Once a position transition is detected, the position classifier developed in Section 5.1 will work to recognize and update the carrying position.

**Figure 4:**
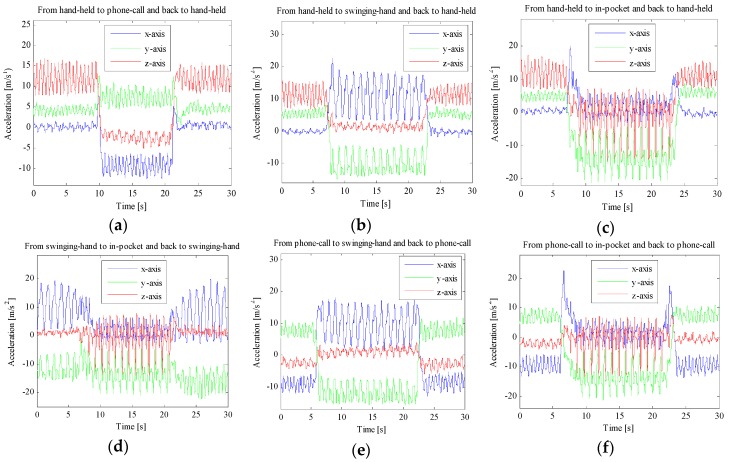
Acceleration signals with all kinds of carrying position transitions: (**a**) between hand-held and phone-call positions; (**b**) between hand-held and swinging-hand positions; (**c**) between hand-held and in-pocket positions; (**d**) between swinging-hand and in-pocket positions; (**e**) between phone-call and swinging-hand positions; (**f**) between phone-call and in-pocket positions.

Our position transition detection algorithm relies on the discovery that, for the duration of a position transition, a rapid and significant change of acceleration signals over three dimensions occurs simultaneously. This may be contributed to the different expressions of the gravity vector caused by carrying position transitions and device orientation changes. Figure 4 shows the three dimensional acceleration signals measured in the device coordinate system with different position transitions. Each sub-figure shows two position transitions: from one carrying position to another and then back to the initial position. A total of six sub-figures show the acceleration signals of all twelve kinds of position transitions. For example, Figure 4a shows the changes of acceleration signals with related position transitions during normal pedestrian walking. The smartphone is held in hand initially for a few seconds; then around 10 s, the device position transits from hand-held to phone-call position; and then this position lasts a few seconds until position transits back to hand-held at about 21 s; finally this carrying positon lasts until the end. 

As can be seen in Figure 4, all kinds of position transitions involve significant changes of the acceleration signals over three dimensions. According to the above discovery, we define two flags for position transition detection. The first flag is used to detect the position transition event, while the second one is used to accurately capture the position transition duration. For the first flag, we exploit the change of the maximum (minimum) acceleration of each signal dimension between consecutive sliding windows, called *cross-window maximum (minimum) acceleration change.* A sum of the cross-window maximum and minimum acceleration changes over three signal dimensions is defined as the first flag, called *cross-window acceleration change*. The second flag is a sum of differences between the maximum and minimum acceleration over three dimensions within a sliding window. For the first flag, we also set the sliding window size of two seconds and with 50% overlap between consecutive windows, the same as in the design of position classifier. This setting guarantees that one position transition duration will be completely included in a sliding window, since one position transition duration always lasts about one second. For the second flag, the sliding window size of one second and 50% overlap between consecutive windows are set to accurately capture the position transition duration.

As seen in Figure 4, a large *cross-window acceleration change and within-window acceleration change* may indicate a present of a positon transition and the specific duration of position transition, respectively. Therefore, we design position transition detection algorithm as follows:

- The cross-window acceleration changes of swinging-hand and in-pocket positions are always larger than those of hand-held and phone-call positions, due to a larger variance of acceleration signals. Thus, we design different thresholds of cross-window acceleration change for different carrying positions.
- For continuous sliding windows, if *cross-window acceleration change Acc*_cw_ exceeds the related threshold *Acc*_th_, a position transition is detected.
- If the *cross-window acceleration change* values of more than two adjacent sliding windows exceed the related threshold, we assume that only one position transition event occurs.
- For the related sliding windows of the first flag exceeding the threshold, we ultimately recognize and capture the position transition duration accurately by selecting the sliding window whose within-window acceleration change is maximal
- Immediately after a position transition and related duration are detected, the position classifier is used to recognize and update the device carrying position.

Compared with the position classifier, the computational cost of the proposed position transition detection algorithm is significantly smaller and can be neglected. For feature extraction, four different features, including the mean, variance, maximum and minimum of related two continuous sliding windows, are required by the classifier, while the detection algorithm only requires the maximum and minimum features. Furthermore, most of time when the carrying position is fixed, the computational cost required by the classification function of a position classifier is avoided. We only require computing the cross-window acceleration change and comparing it with the predefined thresholds to detect the position transitions.

### 5.3. Discrimination between Position Transitions and User Turns

For the proposed position transition detection process, another advantage is to avoid heading estimation errors caused by confusion between position transitions and user turns during pedestrian walking. The PCA-based heading estimation approach only works for a relatively straight walking path, while a large bias may be introduced when a significant user turn occurs due to the corrupted acceleration patterns. Therefore, our previous proposed RMPCA approach combines a user turn detection algorithm to improve user heading estimation. If a user turn is detected, we estimate the user heading by adding the current heading change on the previous user heading.

As in many works [17,31], we identify a user turn by just simply computing the absolute change of device yaw angle *δθ* in the horizontal plane during a sliding window. A user turn is considered to occur when the absolute device yaw angle change *δθ* exceeds a threshold value *δθ*_th_. This algorithm performs well when any position transitions are not considered. Unfortunately, a carrying position transition, which does not actually change the user heading, may generate a relatively large change of yaw angle in the horizontal plane. As a result, it is very likely for a position transition to be considered as a user turn by previous user turn detection algorithms, thus rendering a large heading estimation error.

According to our experiments, though both user turns and position transitions render a relatively large device yaw angle change, the cross-window acceleration change of a user turn is almost the same as that of normal pedestrian walking without carrying position transition. Therefore, we may distinguish between a position transition and a user turn by exploring the cross-window acceleration change. When we find the absolute device yaw angle change exceeds a threshold *δθ* > *δθ*_th_, we also compare the cross-window acceleration change *Acc*_cw_ with the related threshold *Acc*_th_. If *Acc*_cw_ < *Acc*_th_, a true user turn occurs; elsewise, a position transition rather than a user turn is considered to occur. The above discrimination process relies on the assumption that a position transition and a user turn motion don’t happen simultaneously. This assumption is reasonable, since the probability that both motions will happen simultaneously is rather small. According to our experiments in Section 7.1, if the threshold of cross-window acceleration change is chosen appropriately, the position transition detection algorithm can achieve zero false negative and a rather small false positive rates. Therefore, the confusion between position transitions and user turns may be effectively avoided by deploying the proposed position transition detection algorithm.

## 6. User Heading Estimation

**Figure 5:**
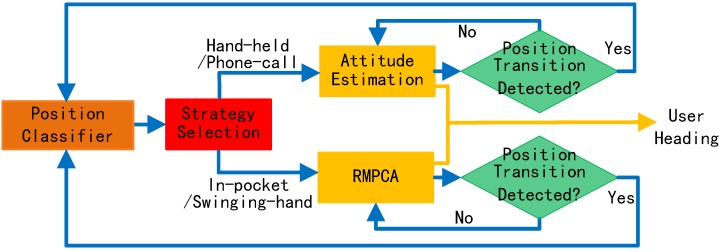
Flow chart of the user heading estimation approach.

The proposed user heading estimation approach consists of two strategies: attitude estimation and RMPCA approaches. The former strategy is selected and used for hand-held or phone-call positions, while the latter one is employed for in-pocket and swinging-hand positions. Figure 5 shows the flow chart of the proposed heading estimation approach. Based on the carrying position determined by a position classifier, the most suitable heading estimation strategy is selected for each carrying position. Meanwhile, a computationally lightweight position transition detection algorithm works continuously. Once a position transition is detected, the position classifier works and the related strategy for heading estimation is selected. Since a compass is almost always unavailable all the time due to the severe indoor magnetic perturbations, we only deploy accelerometers and gyroscopes for heading estimation.

There are two basic assumptions used in the proposed heading estimation approach. First, the smartphone is initially held and gazed at by the user for a few seconds, while the device forward axis is aligned with the user’s walking direction. This assumption is reasonable, since the user needs to gaze at and manipulate the smartphone when he starts the application. Second, we assume that the initial user heading is assumed to be known *a priori*, as in many other works [31,32], which can be obtained by several external positioning systems, such as Global Position System (GPS) tracking when the user enters a building, WiFi localization [33] or landmarks [34]. As a result, the initial user heading and device attitude are all assumed to be known.

Both heading estimation strategies should compute the evolution of device attitude, thus we firstly present a common device attitude estimation module in Section 6.1. Then, we describe the attitude estimation and RMPCA-based approaches in detail in Section 6.2 and Section 6.3, respectively.

### 6.1. EKF Based Attitude Estimation Model

We deploy an EKF based device attitude estimation model by rotation quaternion [35]. In order to describe the rotation quaternion and related rotation matrix, we define the Earth coordinate system as the global coordinate system (GCS) by axes *X_G_*, *Y_G_* and *Z_G_*, which point east, north and in the opposite direction of the gravity vector. Capturing the user heading relies on the outputs of a three-axis accelerometer and three-axis gyroscope built in a smartphone, so we also deploy the device coordinate system (DCS) defined in Section 5.1.

Firstly, we construct the relationship between a rotation quaternion and the smartphone attitude. The transformation from GCS to DCS can be represented as follows:

(1) $$h^{DCS}\left(t\right)={\left(C_{GCS}^{DCS}\left(q\left(t\right)\right)\right)}^{T}h^{GCS}\left(t\right)$$

where $C_{GCS}^{DCS}\left(q\left(t\right)\right)$ is the rotation matrix of DCS with respect to GCS at time t, $h^{GCS}\left(t\right)$ and $h^{DCS}\left(t\right)$ are the 3 × 1 column-vectors at time t relative to GCS and DCS. For simplicity, we will omit the argument t. The rotation matrix can be described by a quaternion:

(2) $$C_{GCS}^{DCS}\left(q\right)=\left[\begin{array}{ccc} q_{0}^{2}+q_{1}^{2}-q_{2}^{2}-q_{3}^{2} & 2\left(q_{1}q_{2}-q_{0}q_{3}\right) & 2\left(q_{1}q_{3}+q_{0}q_{2}\right)\\ 2\left(q_{1}q_{2}+q_{0}q_{3}\right) & q_{0}^{2}-q_{1}^{2}+q_{2}^{2}-q_{3}^{2} & 2\left(q_{2}q_{3}-q_{0}q_{1}\right)\\ 2\left(q_{1}q_{3}-q_{0}q_{2}\right) & 2\left(q_{0}q_{1}+q_{2}q_{3}\right) & q_{0}^{2}-q_{1}^{2}-q_{2}^{2}+q_{3}^{2} \end{array}\right]$$

where $q=\;{\left[\begin{array}{cc} q_{0} & \begin{array}{ccc} q_{1} & q_{2} & q_{3} \end{array} \end{array}\right]}^{T}$ is the normalized quaternion, $q_{0}$ is the scalar part of the quaternion and $\left[q_{1},q_{2},q_{3}\right]$ is the vector part.

Secondly, according to the rigid body kinematic equations [35], the discrete-time model of rotation quaternions can be given as:

(3) $$q_{k+1}\;=\;\exp\left(0.5\times \Omega \left(w_{k}T_{s}\right)\right)q_{k}=\left(I\cos\left(0.5\times \Delta \theta_{k}\right)+\Omega \left(w_{k}T_{s}\right)\sin\left(0.5\times \Delta \theta_{k}\right)/\Delta \theta_{k}\right)q_{k}$$

where $T_{s}$ is the system interval, $q_{k}$ and $q_{k+1}$ are the quaternions at time instants $kT_{s}$ and $\left(k+1\right)T_{s}$ respectively, $w_{k}={\left[\begin{array}{ccc} w_{k}^{x} & w_{k}^{y} & w_{k}^{z} \end{array}\right]}^{T}$ is the angular velocity vector at time instants $kT_{s}$ relative to DCS, I is an 3 × 3 identity matrix, $\Delta \theta_{k}=T_{s}\sqrt{{\left(w_{k}^{x}\right)}^{2}+{\left(w_{k}^{y}\right)}^{2}+{\left(w_{k}^{z}\right)}^{2}}$, and $\Omega \left(w_{k}T_{s}\right)$ is given by:

(4) $$\Omega \left(w_{k}T_{s}\right)=T_{s}\left[\begin{array}{cccc} 0 & -w_{k}^{x} & -w_{k}^{y} & -w_{k}^{z}\\ w_{k}^{x} & 0 & w_{k}^{z} & -w_{k}^{y}\\ w_{k}^{y} & -w_{k}^{z} & 0 & w_{k}^{x}\\ w_{k}^{z} & w_{k}^{y} & -w_{k}^{x} & 0 \end{array}\right]$$

The quaternion $q_{k+1}$ is determined when the initial quaternion $q_{0}$ is known. According to the two assumptions of our heading estimation approach, the initial user heading, device attitude and related rotation matrix of DCS with respect to GCS are assumed to be known. Therefore, the initial quaternion **q**_0_ when a user holds the smartphone and starts the application can be easily computed by deploying the unique mapping relationship between the rotation quaternion and the rotation matrix.

Finally, an EKF is applied to fuse gyroscope outputs with acceleration signals for device attitude estimation. We deploy the smartphone rotation quaternion as a state vector. The state transition equation can be given as:

(5) $$q_{k+1}\;=\;F_{k}q_{k}+w_{k}^{q}$$

where the state transition matrix $F_{k}=\exp\left(\Omega \left(w_{k}T_{s}\right)\right)$, and:

(6) $$w_{k}^{q}=\Xi_{k}w_{k}^{gyro}=-\frac{T_{s}}{2}\left[\begin{array}{c} {}[e_{k}\times ]+q_{0}^{k}I\\ -e_{k}^{T} \end{array}\right]w_{k}^{gyro}$$

$q_{k}=\;{\left[\begin{array}{cc} q_{0}^{k} & \begin{array}{ccc} q_{1}^{k} & q_{2}^{k} & q_{3}^{k} \end{array} \end{array}\right]}^{T}$ is the rotation quaternion at time instants $kT_{s}$, $w_{k}^{gyro}$ is the related white Gaussian measurement noise vector of gyroscope outputs, and $\left[e_{k}\times \right]$ is a standard vector cross-product operator. Equations (5) and (6) are derived from Equation (3), and can be considered as a first order approximation of the “noisy” transition matrix [35]. Consequently, the process noise covariance matrix $Q_{k}$ can be given as:

(7) $$Q_{k}=w_{k}^{q}{\left(w_{k}^{q}\right)}^{T}=\Xi_{k}Q_{k}^{gyro}\Xi_{k}^{T}$$

where $Q_{k}^{gyro}=\sigma_{gyro}^{2}I$ is the covariance matrix of gyroscope measurement noise vector $w_{k}^{gyro}$.

The measurement model is constructed based on the observed acceleration signals:

(8) $$a_{k+1}\;=\;{\left(C_{GCS}^{DCS}\left(q_{k+1}\right)\right)}^{T}g^{GCS}+v_{k+1}^{a}$$

where $a_{k+1}$ and $g^{GCS}$ are the gravity vector at DCS and GCS, respectively, and $v_{k+1}^{a}$ is the related white Gaussian measurement noise. To filter out the signal disturbance caused by a significant body locomotion, we construct an adaptive measurement noise covariance matrix Rk+1=σa2RI:

(9) σa2R={σa2,‖ak+1−g‖2<εa and var(ak+1−Na/2:ak+1+Na/2)<εb∞,otherwise

where $\varepsilon_{a}$ and $\varepsilon_{b}$ define the allowed maximum deviation of acceleration vector from gravity vector and the variance of acceleration signals, respectively, and $\mathrm{var}\left(a_{k+1-Na/2}:a_{k+1+Na/2}\right)$ is the variance of acceleration signals in the sliding window with a size of Na. Thus, the measurement model can be approximated as a linearized formula:

(10) $$a_{k+1}\;=\;H_{k+1}q_{k+1}-H_{k+1}q_{k+1}^{-}+{\left(C_{GCS}^{DCS}\left(q_{k+1}^{-}\right)\right)}^{T}g+v_{k+1}^{a}$$

where $H_{k+1}={\partial a_{k+1}/\partial q_{k+1}|}_{q_{k+1}=q_{k+1}^{-},v_{k+1}^{a}=0}$ is the related Jacobian matrix, $q_{k+1}^{-}=F_{k}{\hat{q}}_{k}$ is the best state estimation of $q_{k+1}$ available, namely the *a priori* state estimate, ${\hat{q}}_{k}$ is the quaternion estimation result of EKF at time instants $kT_{s}$.

Based on the state model in Equation (5) and the measurement model in Equation (10), with the process noise covariance matrix $Q_{k}$ and measurement noise covariance matrix $R_{k+1}$, the EKF model for estimating the state vector $q_{k+1}$ may be established. Detailed procedures for executing the EKF model may be found in [36].

### 6.2. Heading Estimation for Hand-Held and Phone-Call Positions

For hand-held or phone-call positions, we can achieve the user heading estimation by adding the device yaw angle to the related user heading offset:

(11) $$\psi_{user}=\psi_{device}+\Delta \psi$$

where the device yaw angle $\psi_{device}$ can be directly computed by the related quaternion vector as follows:

(12) $$\psi_{device}=\arctan\left(\frac{2q_{0}q_{3}-2q_{1}q_{2}}{q_{0}^{2}-q_{1}^{2}+q_{2}^{2}-q_{3}^{2}}\right)$$

where $q=\;{\left[\begin{array}{cc} q_{0} & \begin{array}{ccc} q_{1} & q_{2} & q_{3} \end{array} \end{array}\right]}^{T}$ is the quaternion estimated by the EKF model presented in Section 6.1. The user heading offset Δψ is the difference between the true user heading and the current device yaw angle. Due to the relatively stable device attitude in the hand-held or phone-call positions, the user heading offset remains fixed until a carrying position transition occurs. Thus, once a position transition is detected, we can reset the heading offset by computing the difference between the true user heading and the related initial device yaw angle of a new carrying position. It should be noted that, the true user heading is assumed to be unchanged during a position transition duration, since the probability of a position transition and user turn happening simultaneously during such a short duration is rather small.

### 6.3. Heading Estimation for In-Pocket and Swinging-Hand Positions

For in-pocket or swinging-hand positions, our previous proposed RMPCA approach is deployed for heading estimation. Due to the rapid heading offset change caused by the highly dynamic body locomotion, the attitude estimation based approach is inapplicable. The fundamental principal of RMPCA is that most of the acceleration signal variations in the horizontal plane will be parallel to the walking direction. Thus, RMPCA extracts the local user walking direction at DCS by deploying the PCA technique. The local user walking direction $WD_{local}$ is obtained by extracting the first principal component of PCA over horizontal acceleration signals. To calibrate the local user walking direction into the GCS, the rotation matrix of DCS with respect to GCS is deployed:

(13) $$WD_{global}=C_{GCS}^{DCS}\left(q\right)WD_{local}$$

where $WD_{global}={\left[WD_{global}^{x},WD_{global}^{y},WD_{global}^{z}\right]}^{T}$, q is the quaternion vector estimated by the EKF model presented in Section 6.1. The user heading estimation can be given as follows:

(14) $$\psi_{user}=\arctan\left(\frac{WD_{global}^{y}}{WD_{global}^{x}}\right)-\frac{\pi }{2}$$

Besides, we also compute the device yaw angle change in the horizontal plane during a sliding window to recognize a user turn. If a user turn is detected as described in the last paragraph of Section 5.3, we estimate the user heading by adding the current heading change on the previous user heading. The RMPCA approach suffers from significant accuracy performance degradation only when a significant user turn occurs. More importantly, the heading estimation error caused by missing a user turn will not accumulate. This is because the local walking direction is extracted independently for each walking step by applying PCA over the horizontal acceleration plane. Rotation matrix mapping of the local walking direction at DCS into GCS is also not affected by missing a user turn. According to our experiments in Section 7.2 for a curved walking path, setting the threshold value of horizontal yaw angle changes at about 20 degrees for user turn detection may obtain the best accuracy performance for the RMPCA approach. More detailed discussions about the choice of threshold value will be given in Section 7.2.

For more detailed descriptions about the RMPCA approach, we refer readers to our previous work [17]. Except for the improved user turn detection algorithm, the main improvement of RMPCA used in our work is that we directly deploy the rotation matrix between DCS and GCS to calibrate the local walking direction into the global direction, rather than deploy an intermediate rotation matrix as in our previous work. This improvement avoids a specific calibration process, and makes the EKF-based attitude estimation model described in Section 6.1 a common module deployed by both the attitude estimation and RMPCA approaches.

## 7. Evaluation

### 7.1. Position Recognition Results

A total number of 4000 sliding window samples from four participants with four different carrying positions were used to train and test the carrying position classifiers. Since different motion states show different acceleration patterns, we designed a specific position classifier for each motion state. By deploying the proposed decision tree-based motion recognition approach, an average recognition accuracy of 99% can be achieved. Therefore, for simplicity, we assume that the user’s motion state is predetermined correctly for each position classifier. We used the 10-fold cross-validation technique to evaluate each carrying position classifier. In 10-fold cross-validation, the data set was divided into ten parts. Out of these ten parts, nine were used for training and the remaining one for testing. This process was repeated ten times by selecting one different part for testing. The position classification accuracy is the average of five different motion states. A five point double move average filter was used to smooth the acceleration signals.

Table 1 shows the position classification results of three compared classifiers including SVM [28], Bayesian Network [29], and Random Forest [30]. The results show that all the three compared classifiers are able to classify the carrying positions with high accuracy. The Random Forest classifier shows the highest average classification accuracy of 97.8%, followed by 96.6% of the SVM classifier and 94.4% with the Bayesian Network classifier. The results in Table 1 show that the in-pocket and swinging-hand positions are the most two confusing classes overall. This phenomenon may be explained by the fact that both the in-pocket and swinging-hand positions show a high variance of acceleration signals due to the dynamic body locomotion. Fortunately, both in-pocket and swinging-hand positions select RMPCA as the most suitable approach for heading estimation, so confusion between the in-pocket and swinging-hand positions does not affect the subsequent selection of an optimal heading estimation strategy. In contrast, a smaller confusion rate between swinging-hand/in-pocket positions and the hand-held/phone-call positions is noted, due to their more distinguishable acceleration patterns.

**Figure 6:**
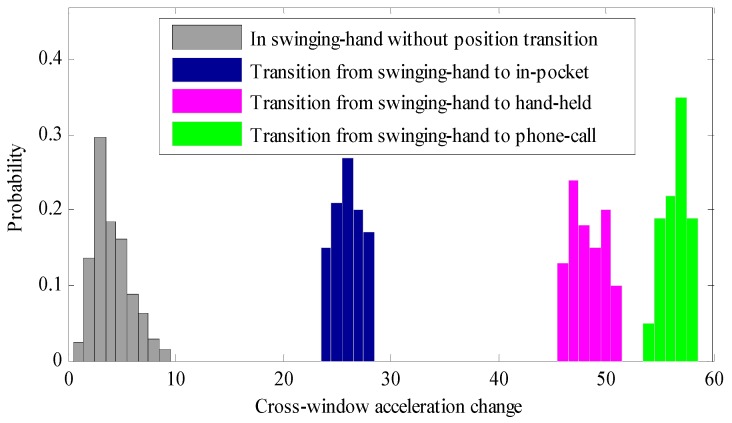
Statistical results of cross-window acceleration change values with and without position transitions for swinging-hand position during normal pedestrian walking.

For the proposed position transition detection algorithm, if the threshold of cross-window acceleration changes is chosen appropriately, the detection algorithm can achieve zero false negative rate and an average 0.6% false positive rate. It is beneficial for heading estimation to reduce the false negative rate as much as possible while keeping a small false positive rate. A false negative case fails to detect the position transition and renders a large heading estimation error caused by rotational movement of position transitions and probable wrong selection of the heading estimation strategy. False negative cases can be always corrected by a position classifier with high classification accuracy. Figure 6 shows the statistical results of cross-window acceleration change values with and without position transitions for swinging-hand position during normal pedestrian walking. We can clearly see that when a position transition occurs, the cross-window acceleration change increases greatly and gives a large enough gap between that it and that without position transitions. The significant increase of cross-window acceleration change values caused by position transitions may also be found with the other carrying positions. In our experiments, for swinging-hand position, the threshold of cross-window acceleration change can be set between 10 and 23. To reduce the false negative rate as much as possible, we can choose a relatively smaller threshold in the allowed interval.

For discrimination between position transitions and user turns, we may also set the related threshold of cross-window acceleration change appropriately to reduce the position transition state false negative rate. This is because, like normal walking without position transitions, the cross-window acceleration change of user turns is also significantly smaller than that when position transitions occur. We studied a total number of 600 position transitions, 100 for position transitions between each pair of carrying positions. A total number of 600 user turns with 150 for each carrying position were also examined. As shown in Table 2, by setting an appropriate cross-window acceleration change threshold, the position transition state false negative rate can be reduced to zero. For user turns, a small probability of 0.02 may be classified as position transition states. However, this can be corrected by a carrying position classifier with high classification accuracy. When the classification result of the carrying position classifier indicates that a position transition does not occur, a user turn may be detected again by comparing the device yaw angle change with the related threshold value. Therefore, the confusion between position transitions and user turns may be effectively avoided.

### 7.2. Heading Estimation Results

**Figure 7:**
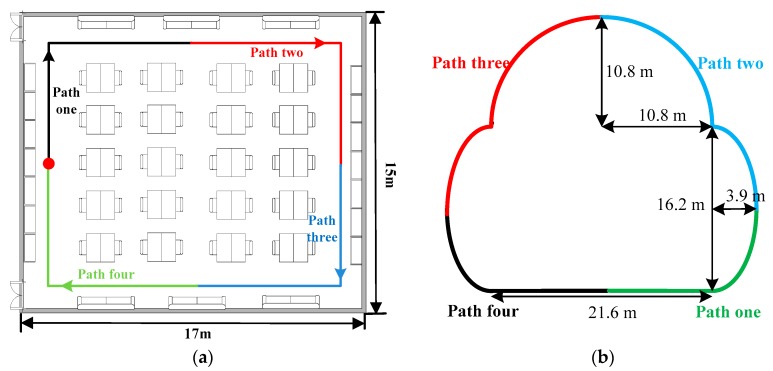
Test environment. (**a**) Environment 1: walking path includes four straight lines and is divided into four sub-paths indicated by different colors; (**b**) Environment 2: walking path includes one straight line, two symmetric parabolas, and a half circle, and is divided into four sub-paths by different colors.

The heading estimation experiments were carried out by four participants in two indoor test environments, as shown in Figure 7. The walking path of Environment 1 includes four straight lines with a total length of 52.8 m. To test the heading estimation approach for non-straight paths, Environment 2 includes one straight line, two symmetric parabolas, and a half circle, with a total length of 76.9 m. Each participant initially held the phone in hand and stood for few seconds to start the application. Before carrying out the experiments, we did all necessary calibrations to make the gyroscope and accelerometer outputs more precise. As in many other works [31,32], the initial user heading is assumed to be known a *priori*. To label the ground truth, we also applied a camera to record the entire walking trajectory of each participant.

The experiments were carried out in two scenarios. In the first scenario, the participant walked along the whole path of the two test environments with a fixed one of the four different carrying positions. In the second scenario, the whole paths of two test environments are both divided into four sub-paths, as shown by different colors in Figure 7, respectively. The participant walked along one of the four sub-paths with one carrying position and then along the next sub-path transited into another position of the remaining three positions with equal probability. Each participant repeated the procedure in the first scenario at least 10 times and in the second scenario 20 times. The average numbers of walking steps required for the total path of two test environments are 88 and 127, respectively. In order to recognize each walking step, we deploy a classical peak detection algorithm [17]. We perform heading estimations for each step, then 3520 and 5080 samples for each position in the first scenario of two environments, 7040 and 10,160 samples in the second scenario of two environments, can be used, respectively. Four heading estimation approaches, including conventional PCA [16], our previous proposed RMPCA [17], uDirect [18] and attitude estimation [13] were compared individually for different carrying positions in the first scenario. The overall performance of the proposed heading estimation approach with position transitions was evaluated and compared in the second scenario.

**Figure 8:**
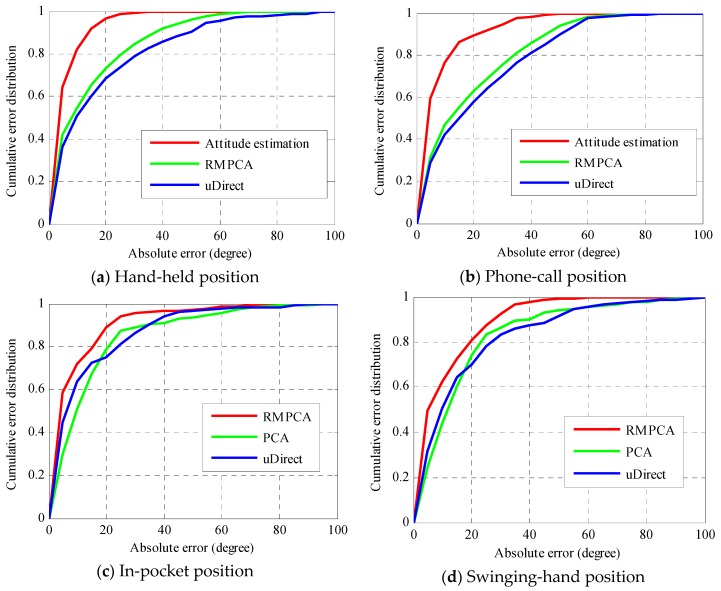
Absolute heading estimation error distributions comparisons of different approaches with four fixed carrying positions in the first scenario of Environment 1.

Figure 8 compares the absolute heading estimation error distributions of various approaches with four different carrying positions in the first scenario of Environment 1. For the hand-held and phone-call positions, the heading estimation performances of RMPCA and PCA are almost the same, since the relatively stable device attitude of these two carrying positions makes both approaches always give the same estimation of horizontal acceleration signals. Thus, for simplicity, we only compare attitude estimation, RMPCA and uDirect approaches, as seen in Figure 8a,b. Clearly, for hand-held and phone-call positions, the attitude estimation approach both achieves significantly better performance than the other compared approaches. For hand-held position, the 50th percentile absolute heading estimation errors of attitude estimation, RMPCA and uDirect approaches are 3.8, 8.6, and 9.4 degrees, respectively, while the 75th percentile absolute errors are 8.2, 22.1 and 26.3 degrees. As seen in Table 3, for the hand-held position, the attitude estimation approach reduces the mean absolute estimation error by 59.4 percent (8.56 degrees), and 67.6 percent (12.22 degrees) than RMPCA and uDirect, respectively. This may be because not only are attitude estimation biases involved in all approaches, but also additional local walking direction extraction biases are introduced by the RMPCA and uDirect approaches. Particularly, for phone-call position, the local walking direction extraction biases introduced by RMPCA and uDirect approaches are more serious, because the required acceleration patterns are always corrupted due to a much smaller magnitude of acceleration signals along the walking direction. Therefore, the attitude estimation approach is the most suitable heading estimation approach for both hand-held and phone-call positions.

For in-pocket and swinging-hand positions, since the attitude estimation approach is inapplicable, we only compare the performances of RMPCA, PCA and uDirect approaches, as seen in Figure 8c,d. Clearly, the RMPCA approach performs significantly better than the other two approaches. For in-pocket position, similar results have been reported in our previous work. For swinging-hand position, a little worse performance than in-pocket position is achieved by RMPCA due to the highly dynamic nature of hand swinging. Nevertheless, RMPCA also performs best. For swinging-hand position, the 50th percentile absolute heading estimation errors of RMPCA, PCA and uDirect are 5.4, 12.1, and 9.7 degrees, respectively, while the 75th percentile absolute errors are 16.6, 20.4 and 23.1 degrees. As seen in Table 3, for swinging-hand position, RMPCA reduces the mean absolute estimation error by 33.8 percent (5.57 degrees), and 36.2 percent (6.19 degrees) than PCA and uDirect, respectively.

Both the PCA and RMPCA approaches employ the PCA technique to extract the local walking direction, which is considered to be parallel to the most variation component of acceleration signals in the horizontal plane. Compared with the PCA approach, the RMPCA approach improves the heading estimation performance by deploying a rotation matrix to achieve a more accurate estimation of the horizontal plane of acceleration signals. For the uDirect approach, the local walking direction is extracted at the moment when the sideway acceleration component is minimized during the walking cycle. Unfortunately, such sideway acceleration components may be always corrupted by hand swinging or leg rotational locomotion, thus some heading estimation errors of large magnitude are introduced by the uDirect approach. Therefore, the RMPCA approach is the most suitable heading estimation approach for both in-pocket and swinging-hand positions.

For Environment 2, RMPCA and the attitude estimation-based approaches still perform best for in-pocket/swinging-hand and hand-held/phone-call positions, respectively. For simplicity, we only give mean and standard deviation of absolute estimation errors in the first scenario of Environment 2, as seen in Table 3. For in-pocket and swinging-hand positions in Environment 2, though most of the walking path is non-straight, no significant accuracy performance degradation is obtained by the RMPCA approach. Mean absolute estimation errors of Environment 2 only increase by 4.5 percent (0.39 degree) and 6.0 percent (0.65 degree) compared to those of Environment 1, for in-pocket and swinging-hand positions, respectively. This is because, for most of non-straight paths with a small curvature, the walking direction may also be accurately extracted by RMPCA, since the walking path for each step may be approximately considered as a straight one. For non-straight paths with a large curvature, added with pedestrians walking swaying sideways, if the user heading change exceeds the related threshold value, a user turn will be considered to occur. The user heading will be estimated by adding the current user heading change to the previous user heading.

In our experiments, setting the horizontal yaw angle change threshold to 20 degrees for user turn detection may obtain the best accuracy performance for the RMPCA approach. Too large a threshold will miss a significant user turns and degrade the accuracy performance. In contrast, too small a threshold may render frequent detection of user turns and even a false detections of straight walking due to sideways swaying motions while walking. In fact, heading estimation by computing user heading change when user turns occur only performs well for a short duration, which cannot adapt the changing device coordinate system for in-pocket and swinging-hand positions. Therefore, a frequent detection of user turns caused by too small a threshold will also degrade the heading estimation accuracy.

**Figure 9:**
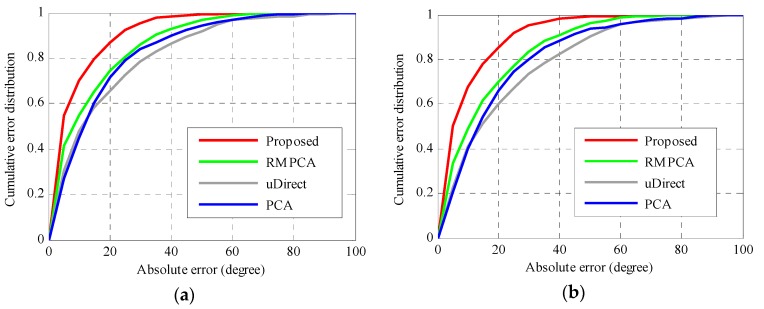
Absolute heading estimation error distributions with carrying position transitions in the second scenario: (**a**) Environment 1; (**b**) Environment 2.

**Figure 10:**
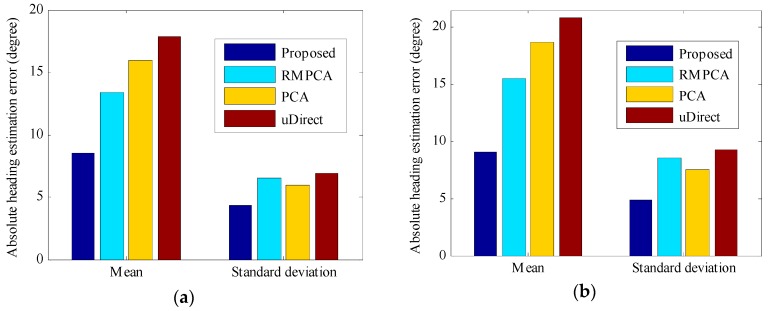
Mean and standard deviation of absolute heading estimation error with carrying position transitions in the second scenario: (**a**) Environment 1; (**b**) Environment 2.

Figure 9 and Figure 10 compare the accuracy performance of different approaches in the second scenario, in which carrying position transitions occur. By deploying the proposed carrying position recognition technique, we can detect the position transition in a timely way and classify the carrying position into the true one. As seen in Figure 9, the proposed heading estimation approach obtains the best accuracy performance, since the optimal strategy is selected for heading estimation of each carrying position. For Environment 1, the 50th percentile absolute heading estimation error of the proposed approach is 4.6 degrees, while those of RMPCA, PCA and uDirect are 8.5, 11.8, and 11.1 degrees, respectively. For Environment 2, the proposed approach also performs significantly better than the other compared approaches, while it performs slightly worse than that in Environment 1, due to the more challenging curved walking path. Furthermore, for all compared approaches, the overall heading estimation accuracy in the second scenario is not the averaged accuracy of the four different carrying positions. Instead, a little worse overall accuracy performance is obtained, since the acceleration patterns required by uDirect and RMPCA approaches are corrupted to some extent around position transition duration. As seen in Figure 10a, the proposed approach in Environment 1 reduces the mean absolute heading estimation error by 36.0 percent (4.82 degrees), 46.3 percent (7.38 degrees) and 51.2 percent (9.24 degrees) compared to RMPCA, PCA and uDirect, respectively. Similarly, as seen in Figure 10b, the proposed approach in Environment 2 reduces the mean absolute heading estimation error by 41.5 percent (6.44 degrees), 51.3 percent (9.59 degrees), 56.4 percent (11.78 degrees) than RMPCA, PCA and uDirect, respectively.

## 8. Conclusions

The knowledge of a user heading can enable many applications ranging from self-localization with PDR techniques to enhanced human computer interaction in smart environments. A major challenge is to allow the user heading estimation to be pervasive for daily use. This paper proposes a novel heading estimation system independent of device carrying positions by exploiting the inertial sensors built into smartphones. By combining a position classifier with a novel position transition detection algorithm, we enable an automatic and accurate detection of carrying position. To reduce computational cost, the position classifier doesn’t work until a position transition is detected by our computational lightweight position transition detection algorithm. Furthermore, the confusion between position transitions and user turns during pedestrian walking, which may render large heading estimation errors, is avoided effectively. Upon the recognized carrying positions, we select the optimal strategy for user heading estimation: attitude estimation approach for hand-held and phone-call positions and RMPCA approach for swinging-hand and in-pocket positions.

Experimental results show that the Random Forest classifier provides the most accurate and reliable carrying position classification with an average accuracy of 97.8%. For the proposed position transition detection algorithm, it can achieve zero false negative rate and a rather small false positive rate, if the cross-window acceleration change thresholds are chosen appropriately. The heading estimation results in the first scenario show that, attitude estimation and RMPCA approaches perform best for hand-held/phone-call positions and swinging-hand/in-pocket positions, respectively. The heading estimation results in the second scenario show that, our heading estimation approach has clear advantages in terms of estimation accuracy compared with conventional individual approaches. Particularly, for Environment 2 with complicated curved walking paths, the proposed approach still performs significantly better than the other compared approaches, while the accuracy performance is only slightly worse than that in Environment 1 with straight paths.

Though we have tested four of the most common carrying positions, we will test some more carrying positions such as in a handbag in our future work. Besides, developing both device orientation and carrying position independent heading estimation systems is an important goal of our future work. For the RMPCA approach, it is already independent of device orientation. For the attitude estimation-based approach, it is a still a challenging problem, because it is difficult to obtain the changing heading offset between yaw angle of device and user heading. We will explore building maps and related landmarks to recalibrate heading estimations and related heading offsets.

## Figures and Tables

**Table 1:** Carrying position classifiers confusion table.

**SVM**	**Hand-Held**	**Phone-Call**	**Swinging-Hand**	**In-Pocket**
Hand-Held	0.972	0.021	0.007	0
Phone-Call	0.029	0.958	0	0.013
Swinging-Hand	0	0	0.981	0.019
In-Pocket	0	0.008	0.040	0.952
**Bayesian Network**	**Hand-Held**	**Phone-Call**	**Swinging-Hand**	**In-Pocket**
Hand-Held	0.945	0.031	0.024	0
Phone-Call	0.020	0.972	0.008	0
Swinging-Hand	0	0.02	0.936	0.044
In-Pocket	0	0.014	0.064	0.922
**Random Forest**	**Hand-Held**	**Phone-Call**	**Swinging-Hand**	**In-Pocket**
Hand-Held	0.990	0.007	0	0.003
Phone-Call	0.018	0.982	0	0
Swinging-Hand	0	0.011	0.969	0.020
In-Pocket	0	0.004	0.025	0.971

**Table 2:** Confusion table for discrimination between position transitions and user turns.

	Position Transition	User Turn
Position Transition	1	0
User Turn	0.02	0.98

**Table 3:** Mean and standard deviation of absolute estimation error (degree) in the first scenario for Environment 1 (E1) and Environment 2 (E2).

**Mean (E1/E2)**	**Hand-Held**	**Phone-Call**	**Swinging-Hand**	**In-Pocket**
RMPCA	14.41/17.43	18.47/22.9	10.91/11.56	8.66/9.05
Attitude Estimation	5.85/6.17	7.83/8.36	\	\
PCA	14.41/17.98	18.61/23.43	16.48/18.21	12.85/13.77
uDirect	18.07/21.97	20.93/26.35	17.10/18.85	13.23/15.22
**Standard Deviation (E1/E2)**	**Hand-Held**	**Phone-Call**	**Swinging-Hand**	**In-Pocket**
RMPCA	5.89/7.95	6.28/8.67	4.86/5.31	4.11/4.42
Attitude Estimation	2.60/2.78	3.71/4.09	\	\
PCA	5.89/8.01	6.36/8.82	5.22/6.16	4.62/4.99
uDirect	6.63/9.14	6.82/9.37	5.91/6.93	5.76/6.97
